# Digital health technologies and innovation patterns in diabetes ecosystems

**DOI:** 10.1177/20552076241311740

**Published:** 2025-02-05

**Authors:** Odile-Florence Giger, Estelle Pfitzer, Wasu Mekniran, Hannes Gebhardt, Elgar Fleisch, Mia Jovanova, Tobias Kowatsch

**Affiliations:** 1 Centre for Digital Health Interventions, 595627 Institute of Technology Management, University of St. Gallen, St. Gallen, Switzerland; 2 Centre for Digital Health Interventions, School of Medicine, 27215 University of St. Gallen, St. Gallen, Switzerland; 3 MTIP, Basel, Switzerland; 4 Centre for Digital Health Interventions (CDHI), Institute for Implementation Science in Health Care, 27217 University of Zurich, Zurich, Switzerland; 5 595497 School of Medicine, University of St. Gallen, St. Gallen, Switzerland; 6 Department of Management, Technology, and Economics, 27219 ETH Zurich, Zurich, Switzerland

**Keywords:** Diabetes, ecosystem, health informatics, digital health technologies, expert interviews, market analysis

## Abstract

**Background:**

The global rise in type-2 diabetes (T2D) has prompted the development of new digital technologies for diabetes management. However, despite the proliferation of digital health companies for T2D care, scaling their solutions remains a critical challenge. This study investigates the digital transformation of T2D ecosystems and seeks to identify key innovation patterns. We examine: (1) What are emerging organizations in digital diabetes ecosystems? (2) What are the value streams in digital T2D ecosystems? (3) Which innovation patterns are present in digital T2D ecosystems?

**Methods:**

We conducted a literature review and market analysis to characterize organizations and value streams in T2D ecosystems, pre- and post-digital transformation. We used the e3-value methodology to visualize T2D ecosystems (RQ1 and RQ2) and conducted expert interviews to identify emerging innovation patterns in digital diabetes ecosystems (RQ3).

**Results:**

Our analyses revealed the emergence of eight organization segments in digital diabetes ecosystems: real-world evidence analytics, healthcare management platforms, clinical decision support, diagnostic and monitoring, digital therapeutics, wellness, online community, and online pharmacy (RQ1). Visualizing the value streams among these organizations highlights the crucial importance of individual health data (RQ2). Furthermore, our analysis revealed four major innovation patterns within the digital diabetes ecosystem: open ecosystem strategies, outcome-based payment models, platformization, and user-centric software (RQ3).

**Conclusions:**

Our findings illustrate the transition from traditional value chains in T2D care to platform-based and outcome-oriented models. These innovation patterns can inform strategic decisions for companies and healthcare providers, potentially helping anticipate new digital trends in diabetes care and across other chronic disease ecosystems.

## Introduction

Diabetes affects approximately 536.6 million people worldwide, with global direct costs estimated at $966 billion in 2021—a staggering 316% increase over the past 15 years.^1^ Almost 90% of global diabetes cases are type-2 diabetes (T2D) cases, a condition in which the body's ability to properly use insulin to process glucose is impaired. Key factors contributing to T2D include modifiable lifestyle behaviors such as physical inactivity and poor diet, as well as genetic predisposition.^1^ Without proper management, T2D can result in persistently high blood sugar levels, leading to serious complications such as nerve damage, kidney failure, and heart disease.^2^

In response to the growing prevalence of T2D, digital health technologies (DHTs) have emerged as valuable tools for managing and preventing T2D in daily life, defined as “computing platforms, connectivity, software, and sensors used for healthcare.”^3^ DHTs, such as continuous glucose monitors (CGMs), can provide patients and healthcare providers (HCPs) with access to continuous and individualized lifestyle and blood glucose levels and help deliver timely lifestyle and disease management recommendations.^4^ These technologies can allow for real-time monitoring and personalized remote care,^4^ thereby potentially supporting both individuals and HCPs in T2D management and prevention, at lower costs.^5,6,7^

As DHTs become more central to diabetes care, they attract increasing attention from tech companies and pharmaceutical firms.^8^ However, beyond the development of DHTs alone, implementing and scaling these technologies across healthcare systems often remains a key challenge.^9^ For instance, healthcare systems are often decentralized and fragmented, lacking coordination among providers, payers, and patients to integrate new technologies efficiently.^9^ Thus, a critical gap remains in understanding the role of DHTs in broader diabetes ecosystems.^10,11^

Prior academic literature on digital technologies in diabetes care has primarily examined the role of DTHs from intra-organizational perspectives, for example by examining individual products, processes, services, or business models in isolations.^10,11^ However, this approach overlooks the broader, inter-organizational dynamics that are essential for understanding the digital transformation of healthcare across multiple stakeholders, including regulators, patients, HCPs, and payers.^12,13^

Examining the role of DHTs T2D ecosystems from an inter-organizational perspective is critical, particularly as in many healthcare systems the lack of integration among stakeholders hinders the scalability of digital health solutions.^14^ To address this gap, our study aims to describe the evolving diabetes ecosystems in light of digital transformation, specifically focusing on the emerging organizations, value streams, and innovation patterns that define this landscape. Given the high prevalence of T2D, this study centers on T2D, though many of the technologies discussed are also applicable to type-1 diabetes (T1D). We specifically pose the following research questions:

1. What are the emerging organizations in digital T2D ecosystems?
2. What are the value streams in digital T2D ecosystems?
3. Which innovation patterns are present in digital T2D ecosystems?

## Theoretical background

### Ecosystems in healthcare and platform ecosystems

Business-related studies^15,16,17,18,19,20^ have identified three major research directions in the field of ecosystems, namely “business ecosystems,” “innovation ecosystems,” and “platform ecosystems.” Most current definitions of ecosystems^15,16,17,18,19,20^ exhibit overlaps, even though they are often formulated in different ways. Within business and innovation research,^21^ the essential components of these definitions encompass four elements, linking three operative concepts—interdependencies, networks, and self-interested actors—with the most common success criterion of an ecosystem: the collaborative value creation of actors in a manner that an individual actor would not be capable of achieving alone.

1. *Interdependencies* ***.*** To accurately depict an ecosystem, it is essential to recognize and analyze all alliances and relationships among the actors. A thorough examination of the intricate interactions within the ecosystem can lead to a more profound comprehension of the fundamental dynamics and dependencies at play.^15^
2. *Network.* The network of an ecosystem is defined by the structure of relationships among its members. Additionally, the network is characterized by members having defined positions and activity flows, and mutual agreement among actors regarding these positions and processes.^15^
3. *Self-interested actors*. In essence, actors can be classified into one of three groups: orchestrators, members, and other organizations. Orchestrators,^22^ also known as architects^18^ cornerstones,^19^ hubs,^20^ or sponsors,^16^ are organizations that possess the capability to both support and derive benefits from the success of an ecosystem through a combination of resources, leadership, and control.^16^ They play a crucial role in consolidating the industry by providing a dominant design for the ecosystem, comprehensively supporting the business model’s value proposition, facilitating collaboration among actors and fostering collective innovation.^19^ The functions of *members* within an ecosystem have been inadequately explored in scientific research thus far. Although members of an ecosystem generally aim to promote the overall success of the ecosystem, their self-interest takes precedence.^23^ Especially in the healthcare field, some providers act in a self-interested way.^24^ In response, policy makers have implemented financial incentives designed to guide providers toward achieving desired outcomes that also reduce costs and enhance efficiency.^25^
4. *Value creation.* It can be stated that the raison d'être of an ecosystem always lies in the fact that actors collectively create value that they would not be able to achieve individually.^26^ An open network prioritizes overall value creation, offering compatibility with various products and established standards. In contrast, a closed network emphasizes individual actor value creation within the same product family, requiring market power and substantial investments.^19^

Specific to healthcare, the role of platform ecosystems is often highlighted.^27^ A platform ecosystem is described as a “Hub-and-Spoke” model, comprising a sponsor with a platform (“Hub”) and providers of complements (“Spoke”), which enhance the value of the platform for consumers.^18,20^ Innovation happens when platform owners want to expand their functionalities to external stakeholders with additional competencies., i.e. connecting disparate stakeholders.^28,29^ Together, platform ecosystems in healthcare are becoming increasingly common as digital technologies become more prevalent.^22,29,30,31,32^

## Methods

In this study, we combine results generated from three different methodologies: a literature review, a market analysis, and expert interviews. We employ data triangulation, a well-established method in qualitative research that enhances validity, robustness, and interpretative potential, while reducing investigator biases and incorporating multiple perspectives.^28,33^ The study was conducted between July 2023 until February 2024 from St. Gallen, Switzerland. A comparison of diabetes ecosystems before and after 2013 highlights not only new stakeholders and value streams but also shifts in innovation patterns. Expert interviews revealed that, alongside the entry of new stakeholders, traditional players adapted their strategies post-2013. We specifically distinguish between traditional (pre-2013) and digital (post-2013) ecosystems due to significant technology adoption impacting diabetes management.

To answer our research questions, we follow the e3-value methodology,^34,35^ building on business and innovation research.^28^ Our method consists of two steps. In step one, we conducted a literature review and a market analysis to identify existing organizations and value streams in the diabetes context, before and after the digital transformation.

*CGM (diabetes-specific innovation)*. The Dexcom G4 Platinum CGM, approved in 2012, was one of the first CGMs to offer real-time glucose readings on a mobile device and set new standards in the accuracy and ease of use. Continuous glucose monitoring (CGM) is seen as one of the most significant breakthroughs in diabetes management in the last 40 years.^36^

*Health information technology (general innovation)*. In the USA, the Health Information Technology for Economic and Clinical Health (HITEC) Act was introduced in 2009, providing financial benefits for HCPs to adopt health information technologies. Between 2009 and 2015, the adoption of physicians of electronic health records jumped from 17% to 78%.^37^ Studies have shown the importance of electronic health records: in many cases health information technology had a positive impact on diabetes care.^38^

*Cloud technology (general innovation).* The adoption of cloud technology had a significant increase of 71% in the year 2012. Cloud computing is essential in diabetes management as it allows individuals to share their glucose data with HCPs in real time.^39^ Also, in academia, there was a significant increase in 2012 with the article number that were published in the field of cloud computing.^29^

*Wearables (general innovation)*. During 2008, the first wireless fitness tracker from Fitbit was launched that could synchronize data with the internet. The Fitbit Flex, released in May 2013, was the first wristband that synchronized with Bluetooth, which allowed users to monitor physical activity levels.^30^ Wearables and, more specifically, fitness trackers are important tools for people living with T2D as they can increase physical activity and reduce the risk of T2D.^31^

**Figure 1.**
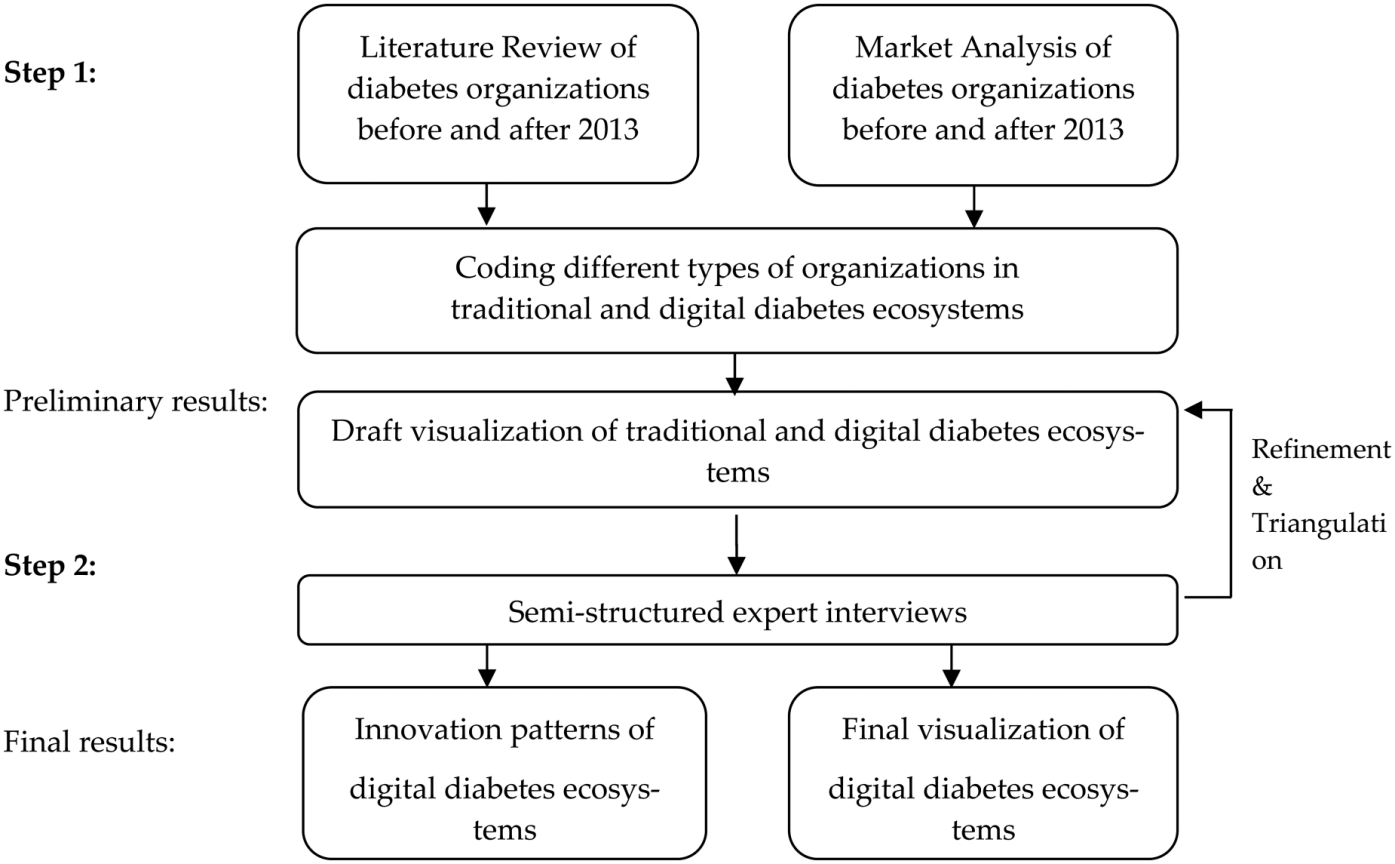
Study workflow and methodological process.

Due to all these reasons, we chose the cutoff at 31 January 2012. Thus, we visualize both ecosystems. In step two, we conducted expert interviews and applied these insights to refine our ecosystem visualizations and derive resulting innovation patterns (see Figure 1 for study workflow).

### Literature review

The literature review aimed to identify emerging organizations and to map out emerging value streams. To this end,^32^ we conducted two separate literature searches, to capture the diabetes ecosystems both (a) before and (b) after the digital transformation, marked in 2013.^40^ The first search included empirical studies until 31 December 2012, while the second search started from 1 January 2013–30 September 2023. Based on business and innovation research,^28^ for traditional diabetes ecosystems, we used the following key terms: ((“Stakeholders” OR “Value network” OR “financial incentives”) AND (“diabet*”)). For digital diabetes ecosystems, we used the following: ((“stakeholders” OR “value network” OR “financial incentives”) AND (“diabet*”) AND (“digit*” OR “innovation”)). The final search included 14 articles for traditional ecosystems and 31 articles for digital diabetes ecosystems. For more details on eligibility criteria, selection criteria of the key terms, data sources, search strategy, and paper selection, see Appendix A in the online supplementary material.

### Market analysis

The goal of the market analysis was to supplement the literature review with additional emerging organizations and value streams, not typically captured in the academic literature.^28^ This means that the study does not rely solely on established knowledge or peer-reviewed studies but also incorporates insights from the real-world marketplace, where new companies and innovations are constantly emerging. By doing this, the study provides a more comprehensive view of the current landscape, capturing cutting-edge trends and developments that may not yet be fully documented in academic sources. This approach ensures that the analysis is more relevant and forward-looking, particularly in rapidly evolving fields like digital health. We conducted a search on diabetes-related companies in Pitchbook, a widely used platform in business science.^41,42^ It is often used in the field of innovation and venture capital.^43,44^ As we aim to find new companies in the field of diabetes, this platform provides a comprehensive overview. Pitchbook enables the identification of organizations associated with diabetes ecosystems as well as new technologies within the field.

Consistent with our literature review, we conducted two separate market searches, to capture diabetes ecosystems both (a) before and (b) after the digital transformation, marked in 2013. We applied the search terms “Diabetes” and “Diabet*” to both traditional and digital diabetes ecosystems. “Diabetes” and “Diabet*” were selected in order to find companies that offer services or products in the field of diabetes as pointed out in the full description of Pitchbook. One limitation is here, that we miss companies that offer diabetes services or products only as a side business. Nevertheless, due to data triangulation with a literature review and expert interviews, we hope to mitigate this bias.

From these searches, we selected the top 100 organizations established before 2013 and the top 100 organizations founded after 2013, based on revenue^45^ with searches conducted on 30 October 2023. By focusing on the top 100 companies with the highest revenue, we aim to ensure that the selected companies have established, proven business models. While this approach may exclude very young and innovative businesses, we chose to prioritize high-revenue companies because most startups (nine out of 10) tend not to survive. Including less established businesses could result in less stable findings.^46^ For more details on company eligibility criteria, see Appendix B in the online supplementary material.

### Literature review and market analyses thematic coding

We used a structured content analysis, including an inductive category development^47,48^ to code the emerging organizations and value streams, resulting from both the literature review and market analyses. We started with the literature review and coded the resulting organizations into pre-defined market segments and generic roles, following business and innovation research.^28^ At the same time, we started to code the organizations from the market analysis with the same approach.^28^ Specifically, for the emerging companies, we followed and adapted the categorization provided by the Digital Therapeutics Alliance, the main international body overseeing digital therapeutic interventions, as relevant to diabetes management.^3^ The three generic roles “DHT—Industry and Admin-facing,” “DHT—HCP-facing,” and “DHT—user-facing” were adopted by the Digital Therapeutics Alliance. When it comes to market segments, only the following were adopted: “digital therapeutics,” “wellness” and “diagnostic and monitoring.” The latter is in the categorization of Digital Therapeutic Alliance separated as two different categories “digital diagnostic” and “patient monitoring.” But as many analyzed companies are to be found in both market segments, we combined these. Other market segments of the Digital Therapeutic Alliance were not used or re-named to make it more specific. Often these are describing a similar concept, namely “Non-Health System Software/DH Solutions,” “Health System Operational Software,” and “Health System Clinical Software, Care Support.”^3^ Following codebook development, two researchers coded organizations into market segments and generic roles, and we assessed coder consistency by calculating Cohen's Kappa. We received a Cohen's Kappa of 0.862, indicating high intercoder reliability.^49^ See Appendix C in the online supplementary material for codebook examples.

Having started the literature review and the market analysis at the same time, we could combine the emerging organizations and value streams into one unified codebook. From there, we drafted the first version of the diabetes ecosystem. In the next step, we sent out the visualization together with the codebook to experts, so that we could adapt the visualization with the value streams and add stakeholders that were not yet found by the literature review and market analysis. In the first part of the interview, we discussed the ecosystem visualization and the codebook with the description of the different market segments. This helped us to detect missing value streams or add additional actors. In the second part of the interview, we asked specific questions about what experts think how the ecosystem has been changed through digital transformation, comparing the years before 2013 and after 2013. There, the goal was to understand more about innovation patterns that arose during that process.

### Ecosystem analysis and visualization with the e3-value methodology

After the literature review and the market analysis, we started to visualize the ecosystem with the e3-value methodology.

To characterize an ecosystem, it is necessary to first identify existing relationships between actors, to better understand their underlying dynamics and dependencies.^15^ Different methods have been proposed to analyze and visualize ecosystems.^50^ Here, we chose the e3-value methodology,^34^ which offers a structured framework for systematic identification, analysis, and visualization of multi-stakeholder relationships in healthcare contexts. The e3-value methodology is particularly well suited due to its conceptual modeling strength in capturing complex, multi-enterprise relationships and economic value exchanges among actors.^34,51,52^ The main aspects of the e3-value methodology are described as actors, market segments, value objects, and value exchange. Actors are autonomous economic and often legal entities recognized by their surroundings. Market segments divide a market into groups with shared characteristics. These actors engage in the exchange of value objects, which can be services, goods, money, or experiences, provided these objects hold significance for at least one entity. Finally, a value exchange connects two actors, reflecting the mutual willingness of actors to exchange value objects.^34,51,52^

### Expert interviews and validation

Expert interviews helped us to provide feedback and validate assumptions about emerging organizations and value streams, derived from the literature review and market analyses.

#### Development of the interview guide

We developed our interview guide based on business and innovation research.^53^ First, we identified the prerequisites for using semi-structured interviews and used our previous knowledge to develop the interview guide. We conducted a literature review on the topic of diabetes management, its stakeholders and its value streams. Also, we read into the theoretical background of ecosystem theories and read gray literature about the dynamics specifically happening in diabetes ecosystems. Besides that, we worked simultaneously on our market analysis to gain an understanding of the most prominent diabetes companies in the field. Second, we formulated the preliminary semi-structured interview guide that consisted of two levels: main themes and follow-up questions. Every participant was questioned on the main themes and there were pre-designed follow-up questions and spontaneous follow-up questions that allowed participants to expand on some particular topics.^54^ Third, we pilot tested the interview guide with a healthcare expert outside of our research team. This was done so that we could assess the appropriateness and comprehensiveness of the interview guide and adapt the wording and arrangements of the questions.^55^

During the interview phase, we adapted the questions several times in an iterative way, as we progressed and got more information about the diabetes ecosystem.

#### Selection of interviewees and procedure

The expert interviews were conducted with healthcare experts of diabetes companies and HCPs using a semi-structured approach.^56^ These individuals were selected for having worked at least 15 years in the healthcare sector, most of them specifically in companies that focus on diabetes management. All of them hold senior to executive roles in their company and were considered as representative of the views of other experts. Information about the professional background of the experts can be found in Appendix D in the online supplementary material. We used purposive sampling in the beginning to select experts, aiming to include professionals from various market segments to provide diverse perspectives on the ecosystems. Purposive sampling is employed to choose respondents who are most likely to provide relevant and valuable information.^57^ It is an effective method for identifying and selecting cases that will maximize the efficient use of limited research resources.^58^ Besides that, we applied snowball sampling, asking the first few participants to recommend others that might fit to our criteria, creating a chain of referrals.^59^ Ten individual one-on-one interviews were conducted until data saturation was reached. Data saturation occurs when the gathered information is sufficient to allow the study to be replicated,^60^ when no new insights can be obtained, and when further coding of data is unnecessary.^61^ Nine interviews were held online via video conference from Switzerland and one in person in Switzerland. However, because the study did not fall under the Swiss Federal Act on Research Involving Human Beings nor raised other ethical concerns, it was exempted from formal review and approval by the Ethics Committee of the University of St. Gallen. We obtained written consent from the interviewees to participate in our study before conducting the interviews.

#### Validation of previous findings

Prior to the interviews, we sent the experts the ecosystem visualization together with the codebook. In the first part of the interview, we discussed the ecosystem visualization and the codebook with the description of the different market segments. There, we asked the experts if they saw any market segments that are missing and if there are any specific emerging organizations that they would want to highlight. Also, we went through each of the value streams to see if we have understood it correctly based on the previous literature review and market analysis and adapted it according to the feedback of the experts. From there, we adapted our ecosystem visualization and codebook iteratively. This helped us to detect missing value streams or add additional actors.

In the second part of the interview, we asked specific questions about what experts think about how the ecosystem has been changed through digital transformation. As all the experts possess at least more than 15 years of experience in the field of healthcare, they all have witnessed the healthcare dynamics before 2013 and after 2013. The experts were asked to compare the different time periods before 2013 and after 2013 in light of digital transformation. This is how innovative patterns could be identified.

#### Coding and derivation of innovation patterns

Based on existing work,^28^ we used a thematic analysis method^62^ to compare traditional ecosystems with digital diabetes ecosystems and specifically derive innovation patterns. We define innovation patterns as new approaches in the current diabetes system that aim to solve reoccurring problems.^63^ Interview-derived innovation patterns help us complement secondary analyses by providing qualitative insights on complex ecosystem changes with potential transferability to other domains.^63–67^

As such, we coded interview responses specific to innovation patterns, following these established steps in the field^62^: (1) familiarization with the data, (2) code generation, (3) theme generation, (4) theme review, (5) theme definition and labeling, and (6) identification of illustrative examples.

## Results

The results are structured along the three research questions: (1) emerging organizations, (2) value streams and (3) innovation patterns in the digital diabetes ecosystem.

### Emerging organizations and value streams in digital diabetes ecosystems

The literature review and the market analysis revealed new organizations from traditional to digital diabetes ecosystems. Here we discuss these organizations (RQ1) with respect to their generic roles and value streams (RQ2). See Table 1 for the generic roles and market segments of the emerging organizations in the current ecosystem, and see Appendix E in the online supplementary material for organizations in traditional ecosystems.

**Table 1.** Emerging organizations in the digital diabetes ecosystem.

Generic Role	Market Segment	Description
Digital Health Technology—Industry and Admin-facing	General definition	Digital health solutions for non-hospital/health system stakeholders (e.g. pharma, MedTech, payors, etc.): clinical administration and management tools, predictive analytics, clinical trial management.^3^
	Real-world evidence analytics	Real-world data and real-world evidence play an increasingly important role in clinical research since scientific knowledge is obtained during routine clinical large-scale practice and not experimentally as occurs in the highly controlled traditional clinical trials. Real-world evidence data can be used for biomarker discovery or validation, gaining a new understanding of a disease or disease associations, discovering new markers for medical stratification and targeted therapies, new markers for identifying persons with a disease, and pharmacovigilance.^72^ *Example:* Roche Diabetes Care^73^
Digital Health Technology—Healthcare provider-facing	General definition	Platforms and health information technology and digital health solutions that support clinicians with managing their healthcare population.^3^
	Healthcare management platform	A healthcare management platform is an online platform designed to enhance collaborative efforts among healthcare providers, facilitating efficient sharing of data.^74^ Furthermore, it can encompass telehealth, which gathers, transmits, and communicates individual's personal health information to their healthcare provider or extended care team, all while being conducted beyond the confines of a hospital or clinical setting, typically in the individual's home.^75^ *Example:* Sirma
	Clinical decision support	A clinical decision support system aims to enhance healthcare delivery by integrating targeted clinical knowledge, patient data, and additional health information to improve medical decision-making.^76^ However, there is a growing trend of developing systems with the ability to utilize data and observations that would otherwise be inaccessible or incomprehensible to humans. Artificial intelligence methods in combination with the latest technologies, including medical devices, mobile computing, and sensor technologies, have the potential to enable the creation and delivery of better management services to deal with diabetes.^77^ *Example:* Glooko^78^
Digital Health Technology—user-facing	Diagnostics & monitoring	Diagnostics: “Validated digital tools for detecting and characterizing disease, measuring disease status, response progression, or recurrence. They are intended to support an individual's self-management of a specific diagnosed medical condition through education, recommendations, and reminders.” ^3^ For example, digital retinal screening tools that are enabled by AI can analyze retinal images to detect early signs of diabetic retinopathy, a common complication of diabetes. ^79^ Monitoring: “Solutions intended to monitor specific health data of individuals that may be used to inform management of a specific disease, condition, or health outcome.”^3^ Commercial diabetes apps offer various self-management features, including logging blood glucose levels, weight, physical activity, blood pressure, and dietary intake. Common features also include educational modules and insulin bolus calculators. Many apps provide feedback through graphical data summaries or phone notifications. Some apps can integrate with blood glucose monitors, export data for sharing, and connect users with healthcare providers for feedback.^80,81^ E.g. digital diagnostics, digital biomarkers, remote monitoring tools, wearables and biometric sensors, medication ingestible sensors *Examples:* BeatO, TwinHealth, Bigfoot
	Digital therapeutics	“Health software intended to treat or alleviate a disease by generating and delivering a medical intervention that has a demonstrable positive therapeutic impact.” ^3^ *Example:* VirtaHealth, Fitterfly
	Wellness	“Disease-agnostic solutions that capture, store, and sometimes transmit health data and promote general well-being and healthy living.”^3^ E.g. Lifestyle and wellness apps (meal tracker, weight tracker, and bolus calculator), activity and fitness trackers, and wearables *Examples:* CashWalk, ModifyHealth
Pharmacy	Online pharmacy	Online pharmacies are businesses operating on the internet that offer pharmaceutical products, including prescription medications, through online ordering and delivery services.^82^ *Examples:* Doc Morris
Community	Online community	In a diabetes online community, members help each other out, educate each other, and share the steps they take every day to stay healthy while living with this very serious condition.^83^ *Examples:* Diabetes Sisters, TuDiabetes

The findings indicate that the digital transformation led to the emergence of three generic organizational roles—DHT (*Industry and Admin-facing, HCP-facing, and user-facing*)—spanning eight market segments: real-world evidence data analytics, clinical decision support, healthcare management platforms, wellness, diagnostics and monitoring, digital therapeutics, online communities, and online pharmacies. While there is not always a one-to-one mapping between each organization, role, and market segment (i.e. companies may simultaneously cover multiple market segments), we follow this framework to synthesize and understand the diverse range of organizations. We explain these emerging organizations in further detail next.

First, *DHT*—*Industry and Admin-facing companies* emerged as important players. These refer to digital health solutions for non-hospital/health system stakeholders (e.g. pharma, MedTech, payors, etc.), namely clinical administration and management tools, predictive analytics, and clinical trial management.^3,68^ For example, Interviewee 8 highlighted this in the following way: “With digital transformation, data analytics is beginning to turn healthcare data into actionable insights, playing a vital role in clinical research and the development of new treatments and drugs*.*”

Second, *DHT*—*HCP-facing companies* primarily address HCPs and manufacturers such as medical device companies and pharmaceutical companies.

These health management platforms play a pivotal orchestrator role by connecting the diabetes ecosystem and bridging various stakeholders, including medical device and pharmaceutical companies, healthcare professionals, patients, and digital health companies. This integration fosters collaboration and enhances the ecosystem's overall value. By integrating different services from different actors, platforms can provide holistic solutions that then might enhance patient outcomes and streamline care delivery. This was stated by Interviewee 2:

Benefits shown in clinical trials cannot be repeatedly shown in real life. Therefore, more services around the glucose value (e.g., CGM), more services around the drug (dosing advice, advice for selecting the drugs which have more targeted benefits), more services around the treatment (when to start insulin, reminder to take the drugs, proposals for exercise) are offered.

Therefore, these platforms often improve their value proposition by leveraging synergies across stakeholders, which was mentioned by Interviewee 2:

The companies that join forces for such an ecosystem can improve their marketing messages since the system can offer more than the individual component.

For example, combining continuous glucose monitoring (CGM) devices with personalized dosing advice and treatment reminders offers a more comprehensive and impactful solution than stand-alone products.

By bringing together diverse offerings, these platforms enhance their appeal to both consumers and HCPs, ultimately improving their market position and effectiveness in diabetes management. This orchestrator role underscores the transformative potential of integrated platforms in creating a more connected and effective diabetes ecosystem.^69^

In another example, software companies like Sirma support HCPs in building up their digital practice through telemedicine or remote monitoring. These companies allow different medical specialists such as clinicians, nutritionists, psychologists, and/or endocrinologists to access the individual's information in real time, particularly as diabetes management requires a comprehensive support from a team of experts (Interviewee 9). By enabling real-time access to information of individuals across different specialties, these platforms streamline diabetes management and promote coordinated care among the individual's entire medical team. Here, value is exchanged by providing software for HCPs and promoting compatibility between disparate stakeholders (Interviewee 5).

Third, when it comes to companies in the field of *DHT*—*user-facing*, numerous companies aim to provide support and guidance directly to users. These emerging organizations, such as digital therapeutics and monitoring companies like the virtual clinic Virta Health, focus on promoting lifestyle changes to manage T2D through personalized healthcare. This was mentioned by Interviewee 5:

The trend that is coming is personalized health care. That's being achieved through adaptive algorithms, essentially artificial intelligence that started off in type 1 diabetes, which is generally where all the technology starts and now also changing the care for people with type 2 diabetes.

Additionally, other companies operate within the wellness domain, like CashWalk, which aims to motivate individuals to increase their physical activity levels. Importantly, these applications are not exclusively for diagnosed users; they can also be utilized by individuals without diagnosis from a preventative standpoint. In these contexts, companies often adopt a direct-to-consumer approach, where customers may pay out of pocket for services.

These wellness and lifestyle technologies sometimes lack comprehensive certification, focusing instead on general health improvements without specific medical claims. The certification process can be complex due to evolving regulatory standards and varying requirements across regions. Many consumer-facing DHTs, which are not subject to the same rigorous certification as medical devices, may pose issues related to data reliability and integration into clinical settings. This lack of certification can hinder their acceptance by HCPs and impact their integration into established medical workflows.^70^

There are also instances, such as, with PatientsLikeMe. This is an online platform where patients connect to share their health experiences, find others with similar conditions, and learn from one another. PatientsLikeMe sells anonymized data from its social media platform to pharmaceutical companies and medical device manufacturers. This data reflects the experiences of platform users regarding their illnesses and treatments, aiding partners in creating more effective and tailored solutions.^71^

### Visualizing organizations and value streams in the digital diabetes ecosystem

**Figure 2.**
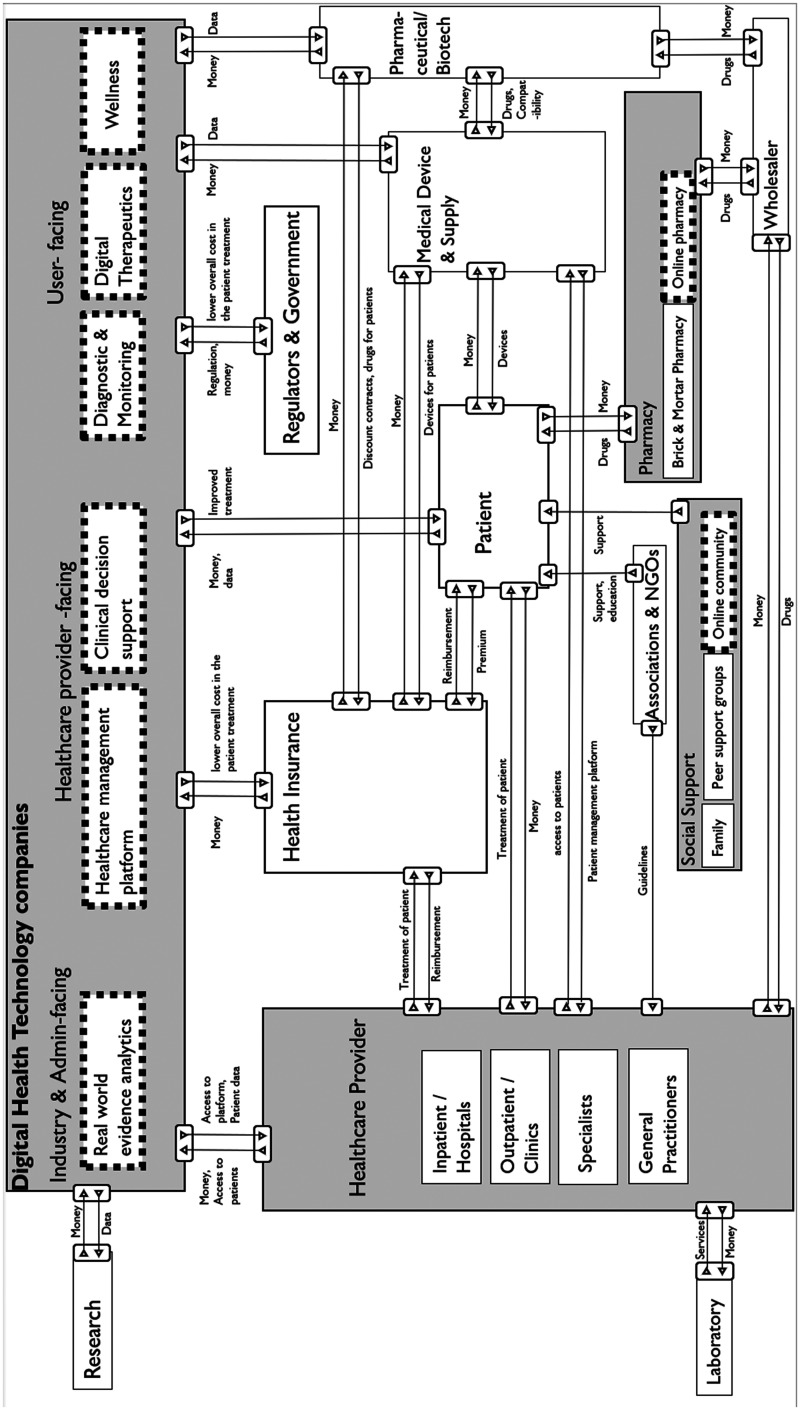
Visualization of digital diabetes ecosystems, derived from literature review, market analyses and expert interviews.

Figure 2 shows the digital diabetes ecosystem, namely connecting DHT companies with additional stakeholders (i.e. individuals, HCPs, health insurances, regulators and government, medical device and supply companies, pharmaceutical and biotech companies, pharmacies, social support groups, laboratories, research centers, and wholesale industries). The general roles of the stakeholders are presented in gray (e.g. *DHT*—*Industry and Admin-facing, DHT*—*HCP-facing, DHT*—*user-facing)*, while the specific market segments are presented in white. Further, the emerging organizations are presented in dotted, white rectangles.

### Four innovation patterns in the digital diabetes ecosystem

A comparison of the traditional and digital diabetes ecosystems before and after 2013 revealed significant shifts in how both new and established stakeholders adapted their strategies. Insights from expert interviews highlighted these changes, which we organized into four key innovation patterns.

Innovation pattern 1 – open ecosystem strategy: *Pharmaceutical and medical device companies must consider participating in an open ecosystem*.

Pharmaceutical and medical device companies apply an open or closed ecosystem approach.

*Sub-theme* – *advantages and disadvantages of an open ecosystem strategy*. In an open ecosystem, the goal is to encourage collaboration with all market participants, ensuring the compatibility of numerous devices with the platform. This enables individuals to utilize devices from various manufacturers, granted these manufacturers have established partnerships with the digital platform (Interviewees 3, 4, 5, and 7). Companies following this approach try to gain market access through different platform collaborations. This approach has two main objectives: firstly, to establish an open ecosystem by offering a variety of devices on the platform, thus promoting widespread adoption among HCPs, and secondly, to boost their marketing of their products within the open ecosystem by influencing preferences toward those products (Interviewee 7). This was mentioned by Interviewee 3:

I would say most of the companies that are device or pharma which have solutions are in the ecosystem to sell their own products.(…) For example, in the U.S., you bring a device to market, and you promote it to many doctors. One of the questions might be, ‘is it compatible with our [company] platform?’

Here, the collectively generated value creation, offering compatibility with various actors, becomes evident.^26^ Additionally, these device manufacturers participate in the open ecosystems of independent health management platforms (Interviewees 2 and 5). The open ecosystem is generally more scalable and contributes to the growth of participating companies (Interviewees 5 and 7). Smaller companies need to join open ecosystems because they lack the influence to independently get HCPs to adopt their platforms (Interviewee 7). However, a disadvantage is the integration costs that the device manufacturer must bear when connecting to an independent platform to ensure compatibility.

*Sub-theme – advantages and disadvantages of a closed ecosystem strategy*. In contrast, medical device and pharmaceutical companies in closed ecosystems create platforms that are only compatible to some degree with other companies (Interviewees 5 and 7).

This was stated by Interviewee 5:

Most of the device companies, pharma companies, to name some, developed their own digital mobile apps. And the problem is that the data is mostly coming from competitors, and they don't always want to share it with one another*.*

Companies adopting a closed ecosystem approach tend to hold significant market share. HCPs assess the value of the ecosystem (Interviewee 6) and have the power to select platforms (Interviewees 3, 4, and 5). When providers utilize a manufacturer's closed platform, they typically limit themselves to that manufacturer's devices, as products from other brands are often incompatible with the platform (Interviewees 3 and 7). Thus, manufacturers actively encourage HCPs to favor and prescribe their devices to strengthen their market position. A benefit of a closed ecosystem is reduced cybersecurity risks (Interviewee 9). However, a drawback is the lack of technological advancement, as independent software companies can concentrate all their resources on enhancing their platforms (Interviewees 3, 5, and 7). Additionally, providing their platform involves a high resource investment (Interviewees 3 and 5).

Both approaches showed advantages and disadvantages. Nevertheless, interviewees largely supported an open ecosystem strategy due to potential benefits for diverse stakeholders.

Innovation pattern 2 – outcome-based payment: *Organizations should be aware of new outcome-based payment as a revenue model and push it further*.

*Sub-theme – real-world evidence data enabling outcome-based payment models*. The evolving healthcare landscape anticipates a discernible shift toward outcome-based payment models as more health data of individuals is collected. In this paradigm, pharmaceutical companies, medical device manufacturers, and HCPs will be required to substantiate the efficacy of their treatments. Using real-world evidence data facilitated by an open ecosystem is pivotal in enhancing treatment approaches. This data not only aids in continually refining treatment methodologies but also positions stakeholders with a stronger negotiating stance.^84^ This forward-looking approach not only underscores the significance of real-world evidence but also emphasizes the strategic advantage in substantiating the value of individuals’ health data and the importance of well-connected platforms (Interviewees 2, 4, 8, and 9).

*Sub-theme*—*challenges with outcome-based payment*. Adopting outcome-based treatment for diabetes is difficult because the disease's complexity makes it hard to clearly determine the effects of different interventions on health outcomes (Interviewee 9). In diabetes ecosystems, coordination incurs costs that prompt a dual assessment—financially and in terms of impact on individuals. The critical inquiry revolves around the cost-effectiveness of coordination efforts, weighing monetary investments against potential advancements. Simultaneously, it questions whether such coordination enhances experiences and outcomes of individuals in diabetes management. Striking a balance between these considerations is crucial to ensure that incurred costs translate into meaningful returns, both financially and in the overall well-being of individuals (Interviewee 7). Another potential risk with new DHTs and medical devices is that they may be marketed to individuals who do not require them. HCPs might adopt the mentality of “If you have a new hammer, every problem looks like a nail.” As mentioned by interviewee 10, who further elaborates:

Progress is great, but it also comes at a cost. If you give for example CGMs, to too many people who don't really need it, it becomes too expensive (e.g., the early stages of Type 2 Diabetes). The danger of ‘one size fits all’ is wrong. Then you feel like you need to give everyone a Freestyle Libre even though it has no benefit. This should also be considered in an ecosystem.

In this case, diabetes ecosystems would get very expensive. Therefore, it was advised to only use new treatment possibilities if it also substantially benefits the individual (Interviewee 10).

Innovation pattern 3—platformization: * Organizations should be aware of the platformization trend, decide on their role in the ecosystem, and prioritize health data as a key resource*.

*Sub-theme*—*data as a key resource for platform companies*. In the context of open ecosystems, platform companies play a crucial role. Tidepool and Glooko are key players in diabetes platforms, with Glooko frequently mentioned in interviews due to its prominence. The company supports data synchronization from over 95% of global diabetes and health monitoring devices, including blood glucose meters, insulin pumps, CGMs, and fitness trackers. Thus, Glooko acts as an orchestrator, connecting HCPs, manufacturers, and individuals.^85^

These platform business models thrive due to the growing interest in health data among stakeholders in the ecosystem. Health data drives innovation in healthcare, as it is considered a key resource in diabetes ecosystems. Analyzing large datasets yields insights that advance medical research, treatment development, and healthcare services improvement. With the help of large data sets, actors can demonstrate the effectiveness of their intervention (Interviewees 2, 8, and 9).

*Sub-theme*—*cybersecurity and transparency as major challenges for platform companies*. While manufacturers strive more and more in this direction, not all HCPs might favor it as they could fear transparent data comparing the effectiveness of treatments of different clinics (Interviewee 9). Interviewee 9 expressed the following viewpoint: “healthcare providers are afraid of transparency. It can reveal inefficiencies and make comparisons possible, which is not always desired.”

Cybersecurity plays a critical role in safeguarding health data, ensuring the integrity of medical records and protecting against the rising threat of cyberattacks. Interviewee 8 mentions: “You have to be very careful to manage the breach of data and of the privacy, that's critical, meaning there is no range of acceptance. You need to grant security.”

The confidentiality and privacy of health information are paramount, and robust cybersecurity measures are essential to maintain trust in healthcare systems (Interviewee 8 and 9). Striking a balance between robust cybersecurity practices and the openness of an ecosystem is crucial to ensure the advancement of healthcare technologies while maintaining the highest standards of data security and privacy (Interviewee 9).

*Innovation pattern 4—user-centric software*. * Organizations creating software must prioritize HCPs and individuals with integration possibilities and user-friendly interfaces*.

*Sub-theme—HCPs and individuals favor user-centric software*. Interviewees concluded that general practitioners, specialists, and other HCPs play a crucial role in the diabetes ecosystem, particularly as they often decide what medical device and drug is most suitable for a given individual (Interviewees 4, 5, and 8).

In the past, HCPs were often swayed to use medications from specific companies through incentives like kickbacks. However, in today's competition-heavy landscape, companies need new strategies to encourage general practitioners to adopt their devices. One common tactic involves user-centric software solutions for health management. Interestingly, there is still ample opportunity for new entrants to the market, especially those offering software with interfaces tailored to the preferences of general practitioners (Interviewees 4, 5, and 8). Generally, it is difficult that HCPs are adopting new technologies. Interviewee 9 mentions the following:

“You have to keep the entry barriers as low as possible and clearly demonstrate the benefits; what's in it for me (healthcare provider) and also show the added value for the care team and patients.”

*Sub-theme*—*HCPs and individuals favor integrative software*. Interviewee 10 mentions the following problem he faces in his daily life:

When patients come to my practice, each of them uses a different medical device from a different manufacturer. I need to log in every time into different systems to see their data. This takes time and is just annoying to remember 10 different passwords for all these systems.

This shows that software must integrate seamlessly with various devices, simplifying the daily workflow for practitioners. Currently, HCPs grapple with many disparate medical devices, used by different individuals. Navigating many different login procedures and interfaces is often described as inefficient, time-consuming, and burdensome. This is why platform solutions that integrate with all different kinds of devices create value for HCPs (Interviewee 10). Thus, to attract HCPs, companies must build user-centric, integrative platforms that are accessible and intuitive (Interviewees 4, 5, 8, and 10).

*Sub-theme*—*consumerization encourages an open ecosystem strategy*. Diabetes companies are also experiencing a shift towards consumerization. This was mentioned by interviewee 8:

When it comes to digital health, the expectation is that people are becoming consumers. So they are committed to sharing the data because they expect that in return of this sharing of data, they will receive some actionable insight they can execute.

Individuals increasingly gather personal data on their wearable devices, such as smartwatches and Fitbits. As data becomes more available in daily life, individuals have more transparency on their disease management and health status (Interviewee 10). With this, there is a need for more personalized, convenient, and accessible data, prompting companies to adopt an open (vs. closed) ecosystem approach and encourage collaboration in response to this evolving landscape (Interviewee 8).

## Discussion

In this study, we conducted a literature review and a market analysis to describe the organizations and value streams in diabetes ecosystems, both before and after the digital transformation. We first demonstrate the emergence of organizations across eight segments within T2D ecosystems: real-world evidence analytics, healthcare management platforms, clinical decision support, diagnostic and monitoring, digital therapeutics, wellness, online community, and online pharmacy (RQ1). We next visualized the value streams between these emerging organizations, highlighting the importance of individual health data (RQ2). Finally, we identified four innovation patterns within T2 diabetes eco-systems (RQ3): open ecosystem strategy, outcome-based payments, platformization, and user-centric software.

### Comparison between traditional and digital diabetes ecosystems

We showed that different market segments within three different generic roles emerged during the digital transformation before 2013 and after 2013. In the *DHT*—*–Industry and Admin role*, real-world evidence companies have become key players. Previously reliant on traditional data collection methods, these companies now utilize wearable devices and mobile health apps for real-time data collection due to digital transformation.^86^ Artificial Intelligence techniques, machine learning, and deep learning are used to analyze unstructured data from sources like electronic health records and social media.^87^

In the *DHT*—*HCP role*, telehealth and clinical decision support systems have advanced significantly. Although telehealth existed before 2013, its adoption has surged due to the COVID-19 pandemic.^88^ Machine learning now supports clinical decision-making for diabetes management.^89^ Clinical decision support systems are categorized into knowledge-based systems, which use predetermined rules, and non-knowledge-based systems, which rely on Artificial Intelligence for actionable insights.^85,90^ Before digital transformation, healthcare systems were fragmented, with poor interoperability and manual processes, leading to inefficiencies and errors.^91^ Today, improved interoperability standards facilitate better integration of healthcare information systems.^92^ Platform companies have become crucial, particularly for automated delivery systems. Significant milestones include the FDA approval of the G4 Platinum CGM in 2013, the Dexcom G5 in 2016, and the Medtronic MiniMed 670G hybrid closed-loop system in 2017.^93–95^

In the *DHT*—*user-facing market*, diabetes monitoring has evolved from manual finger-prick tests to CGMs, which provide real-time glucose data.^95,96^ CGMs are now paired with direct-to-consumer apps like mySugr, which have become popular for their convenience.^97^ However, certification of these apps involves navigating complex regulatory standards, such as the new Medical Device Regulation in the EU, as exemplified by Roche Diabetes Care's efforts to comply.^98^

Regarding innovation patterns, results show that diabetes ecosystems are moving more toward platformization.^99^ This can be confirmed by business and innovation research, which states that the healthcare industry is moving from linear value chains to two-sided markets and, now, to a multi-sided market mediated by platforms. As noted, a platform ecosystem follows a “Hub-and-Spoke” model, where a central sponsor (“Hub”) connects with various complementary providers (“Spokes”). This transition shifts organizations from traditional business models to a platform-based model, enabling value exchange among multiple parties.^18,100^ Today, we are witnessing a rise in interconnected networks of digital technologies, information systems, and processing tools. These networks require a high level of interdependence among competencies and technological complementarity.^101–103^ Nevertheless, this process may be slow due to regulation and the general complexity of the healthcare system. In this context, often the term “value network” is mentioned. This is defined by various interactions where individuals and groups engage with one another in the exchange of values.^104^

Currently, few people with diabetes use platform solutions, but this is likely to change (Interviewees 9 and 10). Such platforms could significantly enhance communication and efficiency between individuals and specialists, providing considerable benefits to users.^104,105^

We found that manufacturers adopted either open or closed ecosystem approaches, each with its own advantages and drawbacks. However, most interviewees favored the open ecosystem strategy for its broader benefits.^106^ Also, a study analyzing ecosystems concluded that the strength of a company does not solely rely on its capabilities but rather on its ability to connect to various competencies. The capability to establish connections with competencies, the fundamental skill of network orchestration, is just as crucial as firms’ specific capabilities.^107^ From the standpoint of a platform company, making decisions about openness and control can be complex, especially in digital health, where several national, regional, and local governmental bodies are involved. Platform companies must strike a delicate balance between leveraging boundary resources to open the platform to external actors and maintaining control over third-party innovations in the periphery. These boundary resources include but are not limited to application programming interfaces, software development kits, contract agreements, app distribution channels, and similar tools that enhance the value for third-party developers.^27^ Sometimes, governments force companies to share their platforms. For instance, the EU is passing new laws, such as the Digital Markets Act, to ensure fair competition online. Apple's App Store is being watched closely for how it controls app distribution. To follow these laws, Apple is changing its App Store rules in Europe, giving users and developers more freedom and access. This change is meant to encourage competition and new ideas in online markets.^108^ To navigate the complex decision-making structure of ecosystems, studies^109^ highlight using a National Architecture Framework as a coordination mechanism. This framework provides application programming interfaces and guidelines to facilitate the development of third-party modules across platform ecosystems. This underscores the multitude of actors in the health domain, spanning both the platform core and periphery.^109^ Especially, the government faces the task of bridging the gap between promoting an open ecosystem for the societal benefits of extensive health data exchanges and ensuring the security and protection of individual health data.^110^ Further, consistent with prior healthcare research,^111^ we found a preference for usability among HCPs or the adoption of systems that are simple to set up and interoperable.

All interviewees emphasized the growing importance of health data as a valuable asset in diabetes ecosystems. Other studies have shown that integrating more healthcare data can create innovation opportunities^,^^112^ such as personalized treatments and improved treatment outcome predictions.^113^ Additionally, applying artificial intelligence to this data can further drive innovation.^114^

However, critical studies^115–117^ raise concerns about the ethical implications of commodifying health information, emphasizing that healthcare innovation operates within an ethically sensitive area. There are concerns about privacy and security, emphasizing the need for stringent measures to protect sensitive health information from breaches and misuse. Additionally, informed consent is crucial; patients must be fully aware of how their data will be used and who will have access to it.^116^ Furthermore, the question of data ownership needs to be clarified to uphold patient rights and maintain ethical standards.^117^

Although the concept of outcome-based payment model is not new,^118^ the implementation of it was developing—depending on the country—rather slow until the last 5 years.^119^ In Europe, the adoption is high in Italy and Spain,^120,121^ whereas in the UK and elsewhere, they still use simple discounting models.^122^ It is described as a type of risk-sharing agreement where payment is tied to the actual results of a product. These contracts allow payers to work with manufacturers to ensure that reimbursements and rebates are based on real-world performance.^123,124^

Further, the collection and analysis of real-world evidence data will continue to expand^,^^125^ driven by the digitalization of healthcare, such as improvements in electronic health records, increased reliance on patient-reported outcomes, and the use of medical monitoring devices (e.g. digital biomarkers).^126^ According to our interview findings, these real-world evidence data play a crucial role in the pharmaceutical industry, helping reduce uncertainties about the long-term effectiveness or cost-effectiveness of new drugs and supporting pricing adjustments. To ensure the benefits and cost-effectiveness of new drugs or therapies, pharmaceutical companies have implemented strategies that foster collaboration with payers through innovative contractual agreements.^127,128^ However, despite these efforts to integrate real-world evidence data, challenges persist in identifying, extracting, analyzing, and validating real-world outcomes.

We contribute to the ecosystem theory in three ways. Firstly, by analyzing diabetes ecosystems, we present empirical evidence of how traditional value chains transform into platforms, connecting different stakeholders. This transformation has also been described by others.^18^ Our findings support existing theory^26^ that ecosystem actors aim to create collective value unattainable individually, as evidenced by medical device and pharmaceutical companies seeking collaboration with DHT companies. Secondly, we enhance the literature of platform ecosystems in healthcare^28^ with empirical evidence of a platformization trend in the field in T2D. We can add to the existing theory that there is a tendency towards an open ecosystem approach that has been pushed by the platformization trend. Lastly, we confirm the theory that digital platforms create value by providing technological components utilized by complementors to create new products or services.^129,130^

For practitioners, leveraging the visualization of diabetes ecosystems can be instrumental in facilitating strategic collaboration options and conducting competitive analyses. This aids in crafting a tailored strategy aligned with the company's objectives. Moreover, it underscores the significance of the platformization trend, prompting thoughtful consideration of how to engage with it effectively. For emerging companies and startups, this ecosystem visualization helps illustrate the diverse incentives of various actors and how they can potentially generate revenue through their digital services. Additionally, the paper enlightens HCPs on their decision to participate in an open or closed ecosystem, emphasizing the impact on the individual. Policymakers and health insurers gain insights into ecosystem dynamics.

### Limitations and outlook

This study has several limitations. First, we have created different generic roles and market segments based on a literature review and a market analysis. Although several authors were involved in that process and validated these categories with interviewees of the diabetes ecosystem, others could end up with a different categorization of these organizations. Second, we assessed 200 companies in the market analysis. Analyzing more companies could bring up a more granular view of the diabetes ecosystems and their market segments. Third, we only focused on English speaking ecosystems in Europe and the USA. Eight out of 10 experts come from Europe. While most interviewees held international positions, experts from other continents and countries might see the diabetes ecosystem differently. Many Asian organizations could not be assessed in detail due to the language used on their website. This means, digital diabetes ecosystems in China might work very differently. Also, even within Europe, some of the value streams might be slightly different depending on the country. Therefore, our visualization cannot be generalized for the entire world. However, this visualization aimed to understand the most prominent dynamics of globally operating organizations from a broader perspective.

Next, we note that although Pitchbook gathers investment data from approximately 3.4 million companies worldwide, the majority (1.8 million) are based in the USA.^43,131^ This imbalance may partly result from higher investment activity in the USA, but it is also likely influenced by Pitchbook's stronger connections with American investment firms and the greater challenges in obtaining data from international investments, particularly in more closed economies. Consequently, despite our efforts to mitigate biases from any single economy, the data we present may somewhat skew towards the dynamics of investments and innovation in the USA.^43^

Exploring the dynamics of open and closed ecosystems could be a promising avenue for future research, unraveling the optimal strategy for different actors in varied contexts. Researchers should investigate crafting an ideal blueprint for open ecosystems, identifying essential components for their success. Additionally, understanding the pivotal role of various actors, such as the state or health insurance, in driving this paradigm is crucial for comprehensive insights. Furthermore, it becomes imperative to ascertain the potential cost savings associated with adopting an open ecosystem approach and thoroughly examine its tangible benefits for the individual's well-being. Lastly, it should be assessed if, in other chronic disease areas, similar ecosystem structures and innovation patterns evolved or will develop soon and to what extent the patterns of diabetes ecosystems are unique.

## Conclusions

Overall, we examined global T2D ecosystems, illustrating the shift from traditional value chains to data-driven platforms and outcome-based payment models in response to digital transformation. Our findings contribute to ecosystem theory and provide strategic insights for diabetes organizations to plan proactively.^132–205^

## Supplemental Material

sj-docx-1-dhj-10.1177_20552076241311740 - Supplemental material for Digital health technologies and innovation patterns in diabetes ecosystems Supplemental material, sj-docx-1-dhj-10.1177_20552076241311740 for Digital health technologies and innovation patterns in diabetes ecosystems by Odile-Florence Giger, Estelle Pfitzer, Wasu Mekniran, Hannes Gebhardt, Elgar Fleisch, Mia Jovanova and Tobias Kowatsch in DIGITAL HEALTH
