# Supplementary material for: Digital health technologies and innovation patterns in diabetes ecosystems
Source: Digit Health. 2025 Feb 5;11:20552076241311740. doi: 10.1177/20552076241311740 (PMC11795620; doi:10.1177/20552076241311740)
Supplement: sj-docx-1-dhj-10.1177_20552076241311740 - Supplemental material for Digital health technologies and innovation patterns in diabetes ecosystems [file sj-docx-1-dhj-10.1177_20552076241311740.docx]

**Collaboration and Innovation Patterns in Diabetes Ecosystems**

**Appendix 1**

## Consolidated criteria for reporting qualitative studies (COREQ): 32-item checklist

Developed from Tong A, Sainsbury P, Craig J. Consolidated criteria for reporting qualitative research (COREQ): a 32-item checklist for interviews and focus groups. International Journal for Quality in Health Care. 2007. Volume 19, Number 6: pp. 349 – 357 [39]

| **No. Item** | **Guide questions/ description** | **Reporting** |
| --- | --- | --- |
| **Domain 1: Research team and reﬂexivity** | | |
| **Personal Characteristics** | | |
| 1. Interviewer/facilitator | Which author/s conducted the interview or focus group? | - Odile-Florence Giger  - Hannes Gebhardt |
| 2. Credentials | What were the researcher’s credentials?  *E.g., PhD, MD* | - Odile-Florence Giger: M.Sc., B.Sc.  -Wasu Mekniran: M.Sc., B.Sc.  - Estelle Pfitzer: M.Sc., B.Sc.  - Hannes Gehardt: B.Sc.  - Mia Jovanova: PhD  - Tobias Kowatsch: Prof.  -Elgar Fleisch: Prof. |
| 3. Occupation | What was their occupation at the time of the study? | - Odile-Florence Giger : Research assistant  -Wasu Mekniran: Research assistant  - Estelle Pfitzer: Research assistant  - Hannes Gehardt: Student research assistant  - Mia Jovanova: Research assistant  - Tobias Kowatsch: Postdoctoral researcher  -Elgar Fleisch: Professor |
| 4. Gender | Was the researcher male or female? | - Odile-Florence Giger: female  -Wasu Mekniran: male  - Estelle Pfitzer: female  - Hannes Gehardt: male  - Mia Jovanova: female  - Tobias Kowatsch: male  -Elgar Fleisch: male |
| 5. Experience and training | What experience or training did the researcher have? | All research team members who conducted the interviews have experience to conduct qualitative research. |
| **Relationship with participants** | | |
| 6. Relationship established | Was a relationship established prior to study commencement? | The research team that conducted the interviews had no prior relationships with most of the participants before the interviews took place. The team had only encountered 2 healthcare professionals before the interviews, whether through university, a research workshop, or personal connections. As a result, the interviewers should be considered unbiased. |
| 7. Participant knowledge of the interviewer | What did the participants know about the researcher? *E.g., personal goals, reasons for doing the research* | The objective of this study was communicated through e-mail. Moreover, the objectives of the study and research purpose were mentioned at the outset of each individual interview. |
| 8. Interviewer characteristics | What characteristics were reported about the interviewer/ facilitator? *E.g., bias, assumptions, reasons, and interests in the research topic* | Information regarding the interviewers’ characteristics and their professional backgrounds was provided on the institute’s website. At the commencement of each interview, during the introduction phase, interviewees were informed of the interviewers’ educational backgrounds and current occupations. |
| **Domain 2: Study design** | | |
| **Theoretical framework** | | |
| 9. Methodological orientation and Theory | What methodological orientation was stated to underpin the study? *E.g., grounded theory, discourse analysis, ethnography, phenomenology, content analysis* | The study was based on a qualitative research methodology conducting semi-structured interviews. We used inductive thematic analysis (please refer to the “Methods” section) |
| **Participant selection** | | |
| 10. Sampling | How were participants selected? *E.g., purposive, convenience, consecutive, snowball* | Participant recruitment and selection were carried out using a combination of diverse sampling approaches. Purposive sampling was employed to encompass a wide spectrum of expertise and functions. Additionally, the snowballing technique was utilized to enhance diversity across demographics, and experiences. |
| 11. Method of approach | How were participants approached? *E.g., face-to-face, telephone, mail, email* | We approached the participants via e-mail. Most of the semi-structured interviews were conducted via video calls, while a portion were in-person. |
| 12. Sample size | How many participants were in the study? | A total of 10 participants took part in the study. |
| 13. Non-participation | How many people refused to participate or dropped out? Reasons? | A total of 4 participants discontinued their involvement in the study. Reasons for dropping out were time constraints or being unreachable after initially agreeing to participate. |
| **Setting** | | |
| 14. Setting of data collection | Where was the data collected? *E.g., home, clinic, workplace* | The interviews were conducted online or at the workplace of the participant. |
| 15. Presence of non-participants | Was anyone else present besides the participants and researchers? | No other individuals were present. |
| 16. Description of sample | What are the important characteristics of the sample? *E.g., demographic data, date* | For specific details about the participants’ professional attributes, please refer to Appendix D. |
| **Data collection** | | |
| 17. Interview guide | Were questions, prompts, guides provided by the authors? Was it pilot tested? | The interview guideline was pilot-tested before the first interview. The participants were informed that no preparation was needed before the interview. The overall interview flow and agenda were explained during the personal introduction. |
| 18. Repeat interviews | Were repeat interviews carried out? If yes, how many? | Two of the interviews were repeated. |
| 19. Audio/visual recording | Did the research use audio or visual recording to collect the data? | All interviews were recorded in audio format following the participants’ consent. |
| 20. Field notes | Were ﬁeld notes made during and/or after the interview or focus group? | No field notes were taken. |
| 21. Duration | What was the duration of the interviews or focus group? | The interviews had an average duration of 58 min. |
| 22. Data saturation | Was data saturation discussed? | The research teams consistently discussed data saturation. |
| 23. Transcripts returned | Were transcripts returned to participants for comment and/or correction? | Interview transcripts were provided to the participants for comment and corrections. Subsequent modifications were made based on their input and feedback. If no response or feedback to the transcript was received within a week, it was regarded as acceptable. |
| **Domain 3: analysis and ﬁndings** | | |
| **Data analysis** | | |
| 24. Number of data coders | How many data coders coded the data? | Data coding was performed by two people. |
| 25. Description of the coding tree | Did authors provide a description of the coding tree? | We have created list of codes and hierarchies of the thematic analysis of the interview data. |
| 26. Derivation of themes | Were themes identiﬁed in advance or derived from the data? | Thematic analysis was employed to identify, analyze, and present first-order concepts, second-order themes, and aggregated dimensions that emerged from the data. |
| 27. Software | What software, if applicable, was used to manage the data? | For data analysis and management, “ATLAS.ti” software was used. |
| 28. Participant checking | Did participants provide feedback on the ﬁndings? | Aggregated dimensions were presented and discussed with two study participants in an online meeting. |
| **Reporting** | | |
| 29. Quotations presented | Were participant quotations presented to illustrate the themes/ﬁndings? Was each quotation identiﬁed? *E.g., participant number* | Participant quotations were presented to illustrate themes and findings. Each quotation is attributed to a participant number ensuring clear identification by the authors. |
| 30. Data and ﬁndings consistent | Was there consistency between the data presented and the ﬁndings? | Yes, there was consistency between the data presented and the findings. |
| 31. Clarity of major themes | Were major themes clearly presented in the ﬁndings? | Yes, the major results were clearly presented in the findings, please refer to “Results”. |
| 32. Clarity of minor themes | Is there a description of diverse cases or discussion of minor themes? | Yes, there is a description of diverse cases and a discussion of minor themes, please refer to “Results” and “Discussion”. |
